# Compressed Sensing Electron Tomography for Determining Biological Structure

**DOI:** 10.1038/srep27614

**Published:** 2016-06-13

**Authors:** Matthew D. Guay, Wojciech Czaja, Maria A. Aronova, Richard D. Leapman

**Affiliations:** 1 University of Maryland, Department of Applied Mathematics and Scientific Computation, College Park, MD 20742, USA; 2 University of Maryland, Department of Mathematics, College Park, MD 20742, USA; 3 National Institute of Biomedical Imaging and Bioengineering, National Institutes of Health, Bethesda, MD 20892, USA

## Abstract

There has been growing interest in applying compressed sensing (CS) theory and practice to reconstruct 3D volumes at the nanoscale from electron tomography datasets of inorganic materials, based on known sparsity in the structure of interest. Here we explore the application of CS for visualizing the 3D structure of biological specimens from tomographic tilt series acquired in the scanning transmission electron microscope (STEM). CS-ET reconstructions match or outperform commonly used alternative methods in full and undersampled tomogram recovery, but with less significant performance gains than observed for the imaging of inorganic materials. We propose that this disparity stems from the increased structural complexity of biological systems, as supported by theoretical CS sampling considerations and numerical results in simulated phantom datasets. A detailed analysis of the efficacy of CS-ET for undersampled recovery is therefore complicated by the structure of the object being imaged. The numerical nonlinear decoding process of CS shares strong connections with popular regularized least-squares methods, and the use of such numerical recovery techniques for mitigating artifacts and denoising in reconstructions of fully sampled datasets remains advantageous. This article provides a link to the software that has been developed for CS-ET reconstruction of electron tomographic data sets.

Electron tomography (ET), as performed in the transmission electron microscope (TEM) or the scanning transmission electron microscope (STEM), has the unique capability of providing three-dimensional ultrastructure of cells and tissues in a native context, thus revealing important constituents such as membranes, cytoskeletal fibers, and protein complexes, on a macromolecular scale. These 3D visualizations are obtained from multiple 2D projections of a biological specimen when it is tilted through a wide range of angles relative to the incident beam direction. Currently, most ET reconstructions of cells are obtained using the weighted back-projection (WBP) algorithm, or the simultaneous iterative reconstruction technique (SIRT) algorithm.

With the continuing development of electron tomographic techniques and their widespread adoption by structural and cell biologists, there has been an impetus to enhance the quality of 3D visualizations to discern smaller structures within the complex milieu of the cell, and to do so with an improved signal-to-noise ratio. Development of more optimal 3D reconstruction algorithms offers one potential route to better visualization of cellular ultrastructure. It has been well established that the quality of a tomographic reconstruction can be improved through the incorporation of prior knowledge about the specimen, i.e. through regularized image reconstruction. More recently compressed sensing (CS), which exploits signal structure to reconstruct a signal from undersampled measurements via regularized recovery, has attracted increasing attention for a number of data processing applications. The success of CS and related mathematical techniques in medical imaging — particularly magnetic resonance imaging (MRI)^1^,^2^,^3^,^4^, and x-ray computed tomography^5^ — have led to a growing interest in using CS methods in the field of electron tomography (ET)^6^,^7^,^8^,^9^,^10^.

Until now, most reports of CS applications in ET have involved the imaging of inorganic materials, e.g. STEM dark-field tomography of nanoparticles^6^,^9^. Recently, however, the CS approach has been applied to STEM dark-field tomography of needle-shaped embedded biological specimens, which are tilted through an angular range of ±90° to avoid missing wedge effects^10^. In addition, there have been studies aimed at applying regularization and CS methods to cryo-TEM^11^,^12^. Here, we consider the application of CS to improve the quality of 3D tomographic reconstructions from microtomed cells and tissues, which have been prepared using heavy atom staining to enhance contrast. This type of specimen preparation technique is the one most widely used among cell biologists because it enables the analysis of large eukaryotic cells. It is well known that compressed sensing reconstruction methods rely on prior assumptions about the statistical properties of the specimen structure being imaged^13^,^14^,^15^,^16^. In this paper, we consider the extent to which the greater structural complexity of biological systems relative to nanoscale materials affects CS-ET performance in these very different categories of specimen.

There is an important distinction between applications of CS methods to MRI and their application to ET, which is crucially important for the mathematical theory of CS-ET. The MRI sampling procedure allows for mechanical modifications to create pseudo-random sampling of a specimen in Fourier space, a process compatible with the theory of randomized sampling in CS. The instrument limitations of ET restrict feasible sampling procedures to projections of the specimen, tilted to an angle within a mechanically allowable range. This has the effect of sampling planes through the origin in Fourier space, a procedure not compatible with any existing CS sampling theory. This obstruction has yet to be adequately resolved and remains an important open problem in the theory of CS-ET. In this paper, we perform numerical simulations of a simple randomized variant of the traditional ET sampling process, wherein projection angles are chosen at random instead of being spaced uniformly in the feasible range. Theoretical obstructions remain for this variant, and simulation results indicate that this method performs worse than traditional tomographic sampling. Thus, there is still a need to develop further mathematical formalism to connect the theoretical foundation with the experimental setting.

The use of scanning transmission electron microscopes (STEM) has become increasingly commonplace in the physical and life sciences for characterizing structures from atomic to macromolecular scales. Under suitable conditions^17^, both bright field (BF) STEM and dark field (DF) STEM measurements may be modeled as linear projections of the 3D electron density in the imaged specimen. Electron tomography can be applied to biological specimens prepared by rapid freezing in a vitrified frozen hydrated state, or by fixation with cross-linking agents followed by plastic-embedding and staining with heavy metals to enhance ultrastructural contrast. The choice of specimen preparation depends on the structures that need to be visualized: whereas it is generally preferable to image bacteria and small eukaryotic cells in a frozen hydrated state using cryo-TEM^18^,^19^,^20^, larger eukaryotic cells and tissues are often best visualized in specimens that have been stained with heavy atom contrast agents, due to limitations in cryo-preparation of large samples^21^,^22^,^23^. ET can be performed on both types of specimen but with different imaging modes: phase contrast TEM for frozen hydrated specimens; and amplitude contrast TEM, bright-field STEM, or dark-field STEM for stained specimens. In this work, we apply CS recovery algorithms to data sets obtained from STEM tomography, which can be applied to thicker specimens and therefore larger 3D volumes^23^,^24^,^25^.

To assess the potential advantage of the technique for determining 3D cellular ultrastructure, we have simulated electron tomographic tilt series from membrane-bound compartments within cells with statistical properties that ensure a high degree of compressibility in the total variation, wavelet, and identity domains, and then perform CS-ET reconstructions from these simulated data sets. We also make comparisons with CS-ET recovery from simulations of nanoscale inorganic materials, and show that the approach gives results that are consistent with previous work by Saghi *et al*.^6^,^9^. Under such conditions, the advantages of CS-ET relative to WBP or SIRT reconstructions are substantial. Reconstructions from a membrane phantom, comprising randomly distributed ellipsoids and spheres at multiple scales, eccentricities, and orientations are used to characterize the performance of CS-ET under a range of noise and measurement conditions. In these simulated membrane reconstructions, we find high a correlation between sparsity and reconstruction error, significant performance advantages of CS-ET relative to WBP, and consistent superiority of uniformly-sampled CS-ET to random-angle CS-ET.

We subsequently analyze experimental STEM tomographic tilt series acquired from plastic-embedded, heavy metal stained sections of fixed cells. Although CS reconstructions substantially outperform the commonly used WBP and SIRT methods for highly sparse objects, our simulations and experimental results indicate that CS generally provides a lesser advantage for reconstructions of biological structures. We attribute this disparity in performance to differences in sparsity between simple phantoms and complex biological structures. We discover that the relationship between data sparsity and the performance of CS reconstruction on the one hand, and between specimen structure and data sparsity on the other, can complicate a thorough understanding of the effectiveness of CS-ET under all potential imaging conditions. However, in application domains for which accurate sparse signal models can be established, there is strong evidence for the efficacy of CS-ET in both fully sampled and undersampled tomogram recovery.

## Results

### Conventional reconstruction techniques in electron tomography

The mathematical theory of linear projections is based on the Radon transform and its relationship with the 2D Fourier transform. Given a 2D electron density function ***f***(x, y) to be recovered from 1D projections, the central slice theorem relates the 1D Fourier transform of the projections to central slices of the 2D Fourier transform of ***f***. The recovery of images via ET can therefore be cast as a problem using Radon transform measurements (real-space methods) or Fourier transform measurements (Fourier methods), and algorithms have been devised for tomographic reconstruction exploiting either or both perspectives. Two algorithms commonly in use today are weighted backprojection (WBP)^26^, and the simultaneous iterative reconstructive technique (SIRT)^27^, and in this work we shall compare the results of the CS-ET technique with those of WBP and SIRT reconstructions performed on tilt series about a single axis. This measurement procedure simplifies the comparative analysis of the three reconstruction algorithms, but since the tilt angles are restricted to a range of ±70° by the specimen geometry in our electron microscope, reconstructions are susceptible to ‘missing wedge’ effects due to loss of information along the specimen’s z-axis. This loss of information can be partially mitigated by collecting tilt series about two orthogonal axes, although we do not consider dual-axis tomography in the present study. If necessary, two such single-axis reconstructed volumes produced by CS-ET, SIRT, WBP, or any other technique can be combined using existing software to produce dual-axis tomograms.

**Figure 1:**
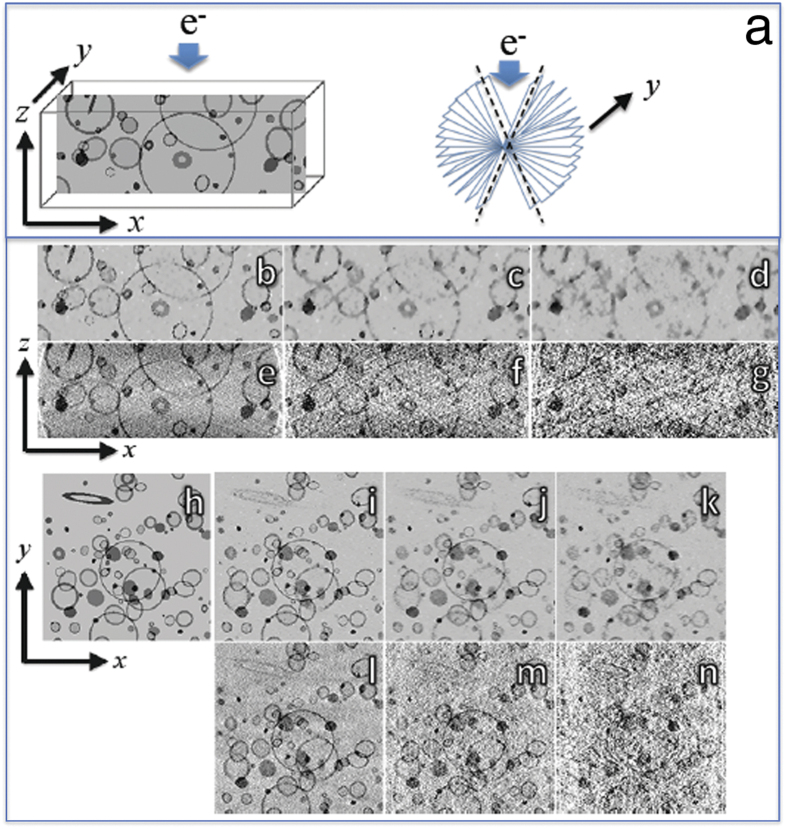
Simulated tomographic reconstructions from a phantom consisting of stained membranes embedded in a uniform matrix. (**a**) Slice through phantom in *x*–*z* plane showing coordinate axis orientation with electron beam along z-axis, and missing wedge of tilt angles indicated by dashed lines; (**b**) CS-ET reconstruction of *x*–*z* slice from simulated projections at ±70° with 2° angular increment; (**c**) CS-ET reconstruction of *x*–*z* slice with 3× undersampling of tilt angles; (**d**) CS-ET reconstruction of *x*–*z* slice with 6× undersampling of tilt angles; (**e**) WBP reconstruction of *x*–*z* slice with all tilt angles; (**f**) WBP reconstruction of *x*–*z* slice with 3× undersampling of tilt angles; (**g**) WBP reconstruction of *x*–*z* slice with 6× undersampling of tilt angles; (**h**) Slice through phantom in *x*–*y* plane; (**i**) CS-ET reconstruction *x*–*y* orthoslice with all tilt angles; (**j**) CS-ET reconstruction *x*–*y* orthoslice with 3× undersampling of tilt angles; (**k**) CS-ET reconstruction *x*–*y* orthoslice with 6× undersampling of tilt angles; (**l**) WBP reconstruction *x*–*y* orthoslice with all tilt angles; (**m**) WBP reconstruction *x*–*y* orthoslice with 3× undersampling of tilt angles; (**n**) WBP reconstruction *x*–*y* orthoslice with 6× undersampling of tilt angles.

In our coordinate system, the specimen is tilted around the y-axis as indicated in Fig. 1a, and the incident electron beam is along the untilted specimen’s z-axis. A detector normal to the electron beam direction captures 2D projections of the specimen’s electron density. These projections are described mathematically as line integrals of the specimen’s electron density along each beam path in the scanning raster. In single-axis parallel beam tomography, the reconstruction of the 3D electron density function ***F***(x, y, z) can be decomposed into independent, parallel 2D reconstructions of slices ***f***(x, y) through the mass distribution function. This reduction of the 3D reconstruction problem into a set of 2D reconstruction problems is convenient both computationally and theoretically. In this paper, we will use ***f*** to indicate the (unknown) electron density function that an imaging specimen possesses, whereas 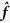 indicates the numerical reconstruction of this density via CS-ET or other computational methods.

### Applying the concepts of sparsity, compressibility and incoherence to CS-ET reconstruction

The ability of CS to recover signals from small numbers of appropriately chosen linear measurements has broadened interest in the technique, which had been mainly applied in the field of medical imaging, including x-ray computed tomography and magnetic resonance imaging (MRI). Recently, Leary *et al*. have applied CS to electron tomographic reconstruction^9^, and here we provide a brief description of the mathematical framework in the context of CS-ET of biological structures, relating to image sparsity, compressibility and incoherence.

Any digital signal can be understood as a vector of data points, and a 2D digital tomographic image N pixels in size can be written as a (column) vector with N entries, each containing a grayscale pixel value. In this paper, we are concerned with the construction of a vector 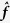, a discrete approximation to the electron density function ***f***(x, z).

The vector 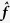 can be represented as a linear combination of basis functions 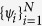:


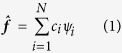


for some length-N vector of coefficients ***c*** = (*c*_1_, *c*_2_, … …. *c*_*N*_), which in CS may be thought of as representation vectors. Given the N × N matrix **Ψ**, where *ψ*_*i*_ is column *i* of **Ψ**, the relationship between 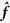 and its transformed representation can be expressed as 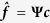. Sparse approximation leverages the observation that, for certain choices of bases **Ψ** and vectors 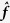 the resultant coefficient vector ***c*** may contain only a few large entries and many negligible entries, so that 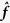 may be well-approximated by a linear combination of only a few of the N basis elements. Common choices appearing in literature include Fourier, discrete cosine and wavelet bases.

A measurement of the density function ***f*** is defined as an inner product 〈***f***, ***φ***〉 with a measurement vector ***φ***, where


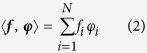


In tomography each measurement is a line integral, and the corresponding measurement vector is nonzero at each pixel of ***f*** through which the projection line passes. A tilt series consisting of τ tilts and σ projection samples per tilt produces a set of T = σ · τ measurements, which can be expressed as a vector ***y*** with T entries.

The measurement vectors 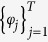 can be ordered as columns in an N × T measurement matrix **Φ**, so that ***y*** = Φ^*^f, where * denotes a conjugate matrix transpose.

The task of CS is to find conditions on ***f***, **Ψ**, and **Φ** that allow for the recovery of ***f*** when 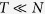. In general, two conditions must be satisfied: first the coefficient vector ***c***, such that ***f*** = **Ψ*****c***, must be sparse; and second, the measurement and representation matrices **Φ** and **Ψ** must be incoherent with respect to each other.

The concept of vector sparsity has a simple definition: a vector ***c*** is sparse if only a few coefficients are nonzero. In practice, signals are rarely sparse, but may possess representation coefficients ***c***, which are compressible, so that only a few coefficients have non-negligible size. Compressible signals are well approximated, though not described exactly, by sparse representation coefficients, and so we will use the term “sparse” to refer also to compressible signals. Compressibility can be measured with an absolute threshold for coefficient size or a relative threshold for coefficient size, which is the measure adopted in the present study. In gauging true sparsity, the proportion of nonzero signal coefficients can be defined as the sparsity ratio of a signal. In gauging compressibility, the proportion of coefficients exceeding a given threshold can be defined as the compressibility ratio of a signal. Thus, a highly compressible signal will have a low compressibility ratio, and vice-versa.

The assumption of signal sparsity is a strong *a priori* restriction on the structure of ***f***, and given an arbitrary unknown vector to be reconstructed there may be little reason to believe that it will have a sparse representation in **Ψ**. Fortunately, suitable representation bases exist for large classes of signals, including many types of images likely to be encountered in tomography.

When ***f*** is itself a sparse vector, we may take **Ψ** to be the identity matrix, and this sparsity can be termed identity sparsity to distinguish it from other types. This is a possible scenario in ET applications where the structure of interest is small in its spatial extent relative to the full field of view. More complex images with features at multiple spatial scales and locations may have sparse representations in discrete wavelet or cosine bases, and for these basis choices the corresponding **Ψ**^***T***^ is a discrete wavelet (cosine) transform. In this work we consider both of these possibilities.

One may attempt to extend the idea of sparse representation further and consider certain nonlinear transforms of ***f***, which result in sparse vectors. One transform commonly used in ET and other image-processing applications is the total variation transform TV[***f***], which measures the magnitude of the gradient of ***f***:


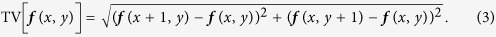


An image having a sparse TV transform contains few pixel-to-pixel variations, dominated by piecewise constant regions with sharp boundaries. This model is appropriate when the information contained in the object of interest consists predominantly of boundaries or other contours. Theoretical results guaranteeing CS reconstruction of TV-sparse objects are scant, but the TV transform’s practical utility and connection to regularized recovery techniques for ET make it an important transform to consider in this paper and our accompanying reconstruction algorithms.

In CS-ET reconstruction it is important to have sufficient incoherence between the measurement vectors, i.e., Radon transform vectors, and also that the measurement vectors be sufficiently incoherence with respect to the sparse representation basis vectors, i.e., low mutual coherence. The coherence of a measurement dictionary containing vectors of length M is defined as √*M* times the largest magnitude among the vectors’ components. An estimate of the coherence between normalized discrete Radon measurement vectors is illustrated in Supplementary Fig. S1 for a 256 × 256 pixel image sampled at tilt angles from −70° to +70° with 5° increments.

At its simplest, the concept of mutual coherence of a representation matrix **Ψ** and measurement matrix **Φ** pertains to the case where {*ψ*_*i*_} is an orthonormal basis and measurement vectors are chosen via a random subsampling of another orthonormal basis {*ϕ*_*j*_}^*N*^ with measurement matrix **Φ**. More general extensions exist^28^, but this basic case is instructive for understanding how representation and measurement choices affect CS-ET.

The mutual coherence *μ*(**Φ**, **Ψ**) between a measurement vector and a representation vector is defined as the largest inner product of any *ϕ*_*j*_ with any *ψ*_*i*_:


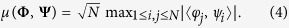


In other words, mutual coherence is the magnitude of the largest correlation between a measurement vector and a representation vector. The mutual coherence of two orthonormal bases is constrained to lie in [1, √*N*], and the bases are incoherent if *μ* is close to 1. The Euclidean and Fourier bases are an example of a pair of maximally incoherent bases, with *μ* = 1. Intuitively, two bases are incoherent if no element of one has a concentrated representation in the other. An estimate of the mutual coherence between normalized discrete Radon measurement vectors sampled at tilt angles from −70° to +70° with 5° increments, and elements of a BD8 wavelet basis for 256 × 256 pixel images and sampling 10% of the vectors in each dictionary is illustrated in Supplementary Fig. S2.

Given this definition, a hallmark result in CS theory^29^ states that a signal with *k* non-zero representation coefficients in basis **Ψ** can be recovered from *m* randomly-selected measurement vectors in **Φ**, where


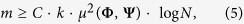


for some small constant C. The value *k* indicates the sparsity level of the input signal. Importantly, this expression is linear in the sparsity of the signal to be recovered, and only logarithmic in the size of the signal. In this regard, CS is applicable when the measurement vector and the representation vector are incoherent, i.e., *μ*(**Φ**, **Ψ**) has a sufficiently small magnitude, and the signal is sparse 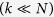.

In order to recover ***f*** from these few coherent measurements, it is necessary to solve a nonlinear convex optimization problem. A key result in CS theory states that, for a sufficiently sparse signal ***f*** and low coherence between measurement and representation matrices **Φ** and **Ψ**, we can recover ***f*** from the measurements, ***y*** = **Φ**^***T***^***f***, by finding the unique minimizer of the equation


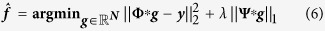


where *λ* is a regularization hyperparameter, 
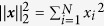
 is the square of the Euclidean vector norm, and 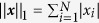 is the 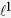 vector norm^29^.

This equation can also be understood as the problem of recovering ***f*** from ***y*** using a regularized least-squares approach^30^. Most generally, regularized least-squares methods encompass a broader range of equations than this, but equations of this form appear in ET regularized recovery literature^31^. The distinction between CS and regularized least-squares recovery is in the design of the measurement matrix not in the choice of numerical technique used for image reconstruction. Therefore, for 3D reconstructions from tilt series acquired with the standard constant tilt-angle increment, CS-ET is, in fact, equivalent to regularized least-squares image recovery.

The regularized recovery approach also allows for the imposition of more than one penalty term to improve reconstruction accuracy, a modification to (6) that is more difficult to justify through CS theory^10^. The full equation to be minimized in this work is therefore


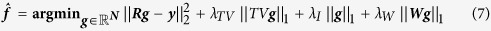


where matrix ***R*** has Radon measurement vectors as its rows, ***W*** is a DB8 wavelet transform^32^, and (*λ*_*TV*_, *λ*_*I*_, *λ*_*W*_) is a collection of regularization hyperparameters for TV, identity, and wavelet transforms, respectively. By exploiting the Fourier Slice Theorem, one may treat STEM tilt series data as samples in either the Radon or Fourier domain^33^. Previous implementations of CS-ET have been done in the Fourier domain^6^,^9^,^10^,^34^,^35^, requiring additional numerical approximation and the computation of NUFFT operators, which are considerably slower than the familiar FFT operator^36^. We have therefore chosen to work in the Radon domain with the MATLAB platform using a graphical processing unit (GPU) on a PC computer. More details about the algorithm and its implementation are described in the Methods. Although our reconstruction was done in the Radon domain, the overall methodology closely parallels the development described by Leary *et al*.^9^.

### CS-ET reconstructions from simulated tilt series

We first applied the CS-ET reconstruction technique to a tilt series of simulated dark-field STEM images of circular and crescent-shaped objects, as from a cross-section of inorganic nanoparticles. This model of simple, high-contrast structures reflects the image statistics present in certain materials science applications of STEM tomography (Supplementary Fig. S3a). As shown in Supplementary Fig. S3b–j, the resulting CS-ET reconstructions are significantly clearer than their SIRT and WBP counterparts, in agreement with the previous work of Leary *et al*.^9^. This type of simple nanoparticle specimen is ideal for application of the CS reconstruction approach; and the results show that our implementation of a real-space CS algorithm gives the expected result.

For values of k = 1, 3, and 6, we created k× undersampled tilt series by keeping every kth projection from an experimental or simulated full tilt series. To avoid this cumbersome language, we instead refer to these tilt series as “k× undersampled”.

We next tested the CS-ET technique on a simulated tomographic STEM bright-field tilt series generated from a 3D phantom imitating a collection of stained membrane-bound cellular compartments. 2D slices of this phantom are shown across the *x*–*z* plane in Fig. 1a, and across the *x*–*y* plane in Fig. 1h. Simulated projections were acquired independently for each *x*–*z* slice and consisted of Radon transform data over a tilt range of ±70° with an angular increment of 2°. As described in the Methods, Poisson noise simulating typical high-dose STEM measurement conditions was added to the projections. The projections were then further corrupted with Gaussian noise. The membrane phantom *x*–*z* plane reconstructions were performed with noisy 1×, 3×, and 6× undersampling of the tilt angles, using CS-ET (Fig. 1b–d) and WBP (Fig. 1e–g). Corresponding *x*–*y* plane reconstructions are shown in Fig. 1i–k for CS-ET, and in Fig. 1l–n for WBP. Results from noiseless membrane phantom reconstructions are shown in Supplementary Fig. S4. It is evident that the performance of CS-ET exceeds that of WBP for the fully sampled and undersampled tilt series, but the advantage of CS is greatest for the 6× undersampled tilt series. This observation is corroborated in Supplementary Fig. S5, which displays RMSE calculations for both reconstruction methods. These results are in agreement with known results for CS recovery of sparse signals from undersampled data. However, the advantage of CS relative to WBP is not as significant for the membrane phantoms as it is for the nanoparticle phantom.

**Figure 2:**
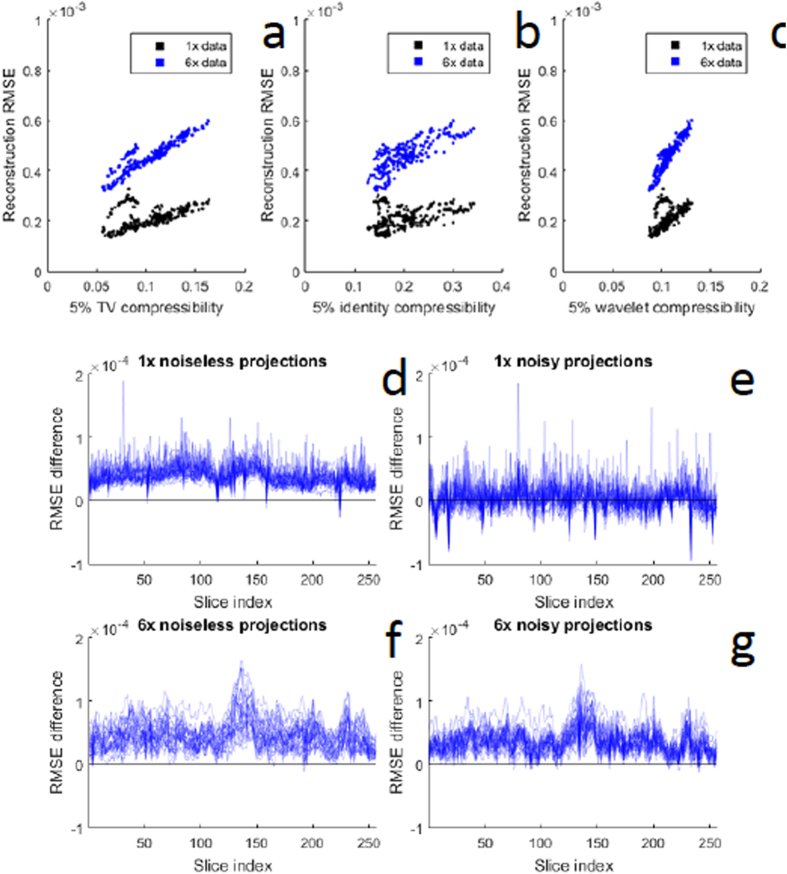
Root mean square errors (RMSE) in successive slices of CS-ET reconstruction calculated from membrane phantom. (**a**–**c**) Correlation between RMSE and compressibility ratio for CS-ET reconstructions of the membrane phantom. The n%-compressibility ratio of an image is defined as the proportion of pixels with magnitude larger than n% of the maximum-magnitude pixel’s magnitude. This can be calculated in any transform domain by applying the same procedure to the transformed image. The higher the compressibility ratio, the less compressible is the image. Plots show RMSE versus 5%-compressibility ratio for (**a**) TV transform, (**b**) identity transform, and (**c**) DBS wavelet transform. (**d**–**g**) Comparison of reconstruction errors from simulated STEM tomographic data based on uniform versus random sampling of tilt angles. A simple variant of conventional, uniformly sampled tomographic measurement is to choose tilt angles at random within the mechanically feasible range. Numerically, this approach performs worse than uniform sampling for the membrane phantom. For the two membrane phantom reconstructions from 1× fully sampled, and 6× undersampled tilt series, 30 random reconstructions were performed using the same number of tilts at angles but chosen at random between −70° and 70°. This procedure was repeated using both noiseless and noisy projection data. The difference between RMSEs of 30 random-angle reconstructions and 30 uniform-angle reconstructions are plotted for the 1× trials with and without noise in (**d**,**e**), respectively, for 256 *x*–*z* reconstruction slices, indexed along the *x*-axis; and for the 6× trials with and without noise in (**f**,**g**), respectively. A positive RMSE difference indicates that random sampling performed worse than uniform sampling.

We expect that the advantage of CS methods for reconstructing sparse objects in electron tomography should be manifested in a correlation between the root mean square error (RMSE) and the compressibility of each of the different *x*–*z* planes in the reconstruction. We measure compressibility as an n%-compressibility ratio, defined as the proportion of image pixels with magnitude greater than n% of the largest magnitude pixel in the image. The more compressible an image, the smaller is the compressibility ratio. This relationship is shown in Fig. 2a–c for the membrane phantom, where we calculate a 5% compressibility ratio in the three sparsity domains analyzed in this study (TV, identity, wavelet). In all three transform domains, there is a clear positive correlation between reconstruction error and compressibility ratio for fully sampled and 6× undersampled tilt series. This demonstrates the importance of signal sparsity for CS reconstruction methods.

We then compared reconstructions obtained using a constant tilt angle increment, with reconstructions obtained by creating projections at randomly sampled angles over a tilt range of ±70°, to test whether there was any improvement by decreasing the mutual coherence between the measurement basis functions and the representation basis functions of the object. However, our numerical results shown in Fig. 2d–g indicate that this sampling strategy performs worse than traditional uniform sampling. Since CS performance is dependent on signal sparsity, we quantify this phenomenon by comparing the sparsity of the nanoparticle and membrane phantoms in the TV, identity, and wavelet domains in Supplementary Fig. S6. It is evident that the membrane phantom is substantially less sparse than the nanoparticle phantom.

### CS-ET reconstructions of experimental tomographic tilt series

To test the CS-ET reconstruction algorithm on experimental data, we recorded STEM tomographic tilt series from insulin-secreting beta cells in a specimen of isolated mouse pancreatic islets of Langerhans, which was conventionally prepared for electron microscopy by fixation with osmium tetroxide, dehydration, embedding and staining with uranium and lead^25^,^37^. Cellular ultrastructure prepared in this way is rich in stained membranes, which are visible as dark lines in bright-field STEM images or bright lines in dark-field STEM images. Islet beta cells contain many internal structures of this type, including outer membranes of secretory granules, nuclear inner and outer membranes, mitochondria, and endoplasmic reticulum. In addition, there are many other structures such as ribosomes, cytoskeletal filaments, and other macromolecular assemblies. The sample represents a complex biological object on which different reconstruction techniques can be tested. Single-axis tilt series were reconstructed as 1024 independent x–z slices, perpendicular to the y-axis, each containing images of size 1024 × 100 pixels, using CS-ET, SIRT, and WBP for fully sampled tilt series as well as 3× and 6× undersampled tilt series.

**Figure 3:**
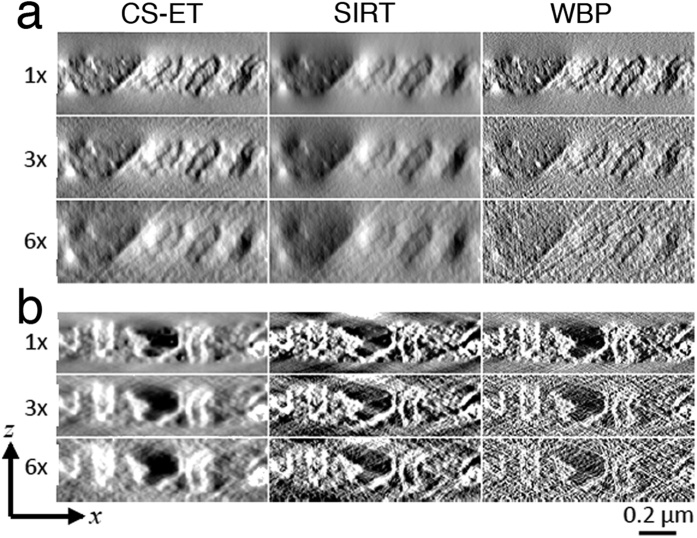
Comparison of CS-ET, SIRT, and WBP reconstruction *x*–*z* slices through mouse beta cell. (**a**) Reconstructions from high-dose bright-field STEM tilt series, (**b**) reconstructions from high-dose dark-feld STEM tilt series.

The tomographic reconstructions in the *x*–*z* plane from tilt series acquired in bright-field STEM and dark-field STEM in Fig. 3a,b, respectively, indicate that there is a significant reduction of noise in CS-ET and SIRT reconstructions relative to WBP. Corresponding reconstructions in the *x*–*y* plane are displayed in Supplementary Fig. S7, for the bright-field STEM dataset, and in Supplementary Fig. S8 for the dark-field STEM dataset. The distinction between CS-ET and SIRT reconstructions is less pronounced, though SIRT structures overall exhibit some blurring that is less apparent in CS-ET results.

**Figure 4:**
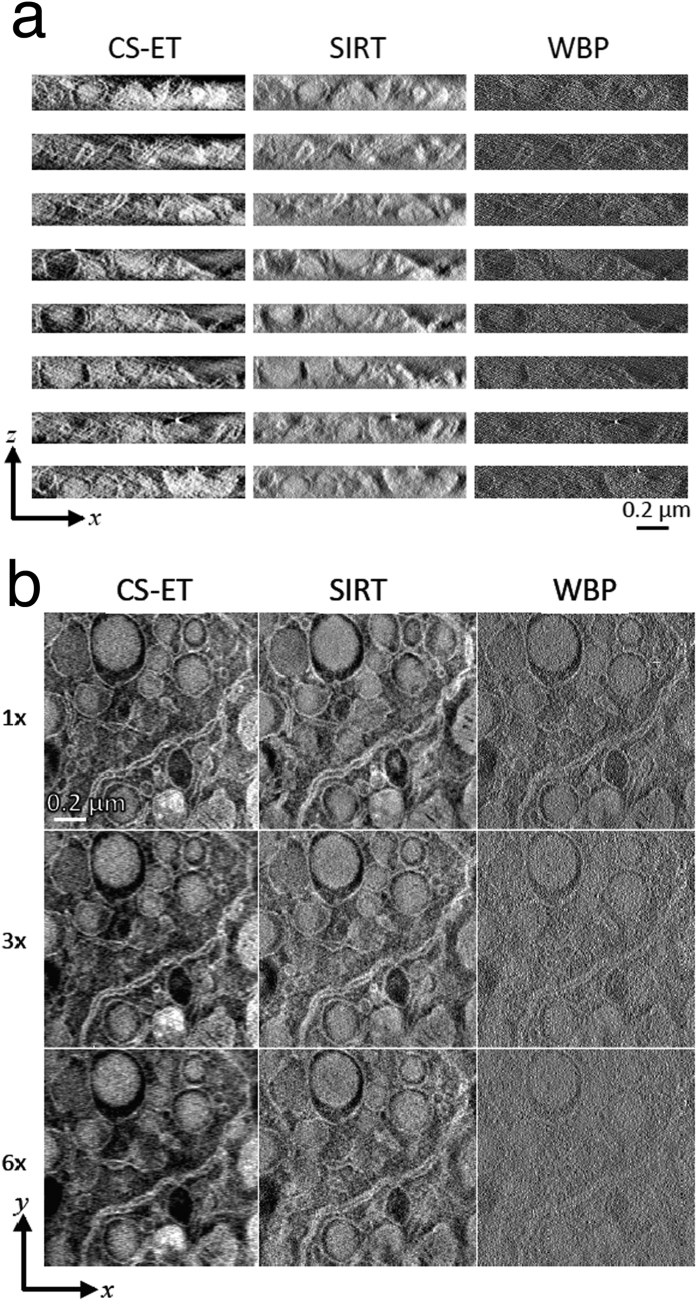
Comparison of CS-ET, SIRT, and WBP reconstruction algorithms from dark-field STEM tilt series of mouse beta cells recorded at low electron dose. (**a**) Reconstructed *x*–*z* slices, (**b**) reconstructed *x*–*y* slices from fully sampled, 3× undersampled, and 6× undersampled tilt series. Note that due to differing reconstruction procedures, a small discrepancy exists in the z coordinates of the CS-ET and SIRT *x*–*y* slices, manifesting as small structural differences between the images.

Next, we compare CS-ET, SIRT, and WBP reconstructions from dark-field STEM tilt series recorded at low electron dose. The eight *x*–*z* slices in Fig. 4a indicate that both CS-ET and SIRT perform substantially better than WBP, and again CS-ET structural boundaries appear less diffuse than their SIRT counterparts.

In Fig. 4b, we compare CS-ET, SIRT, and WBP reconstruction *x*–*y* slices from fully sampled, 3×, and 6× dark-field STEM tilt series recorded at low electron dose. From this perspective, the advantages of the CS-ET algorithm are more apparent. The WBP reconstruction becomes heavily degraded by noise with increased undersampling. A significant portion of this noise is absent from the SIRT reconstructions, but CS-ET exhibits still lower noise levels in the 3× and 6× undersampled reconstructions.

### Comparisons of sparsity between experimental and simulated data

**Figure 5:**
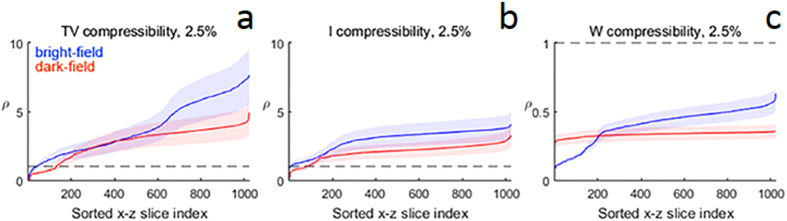
Compressibility of CS-ET reconstructions relative to the membrane phantom. 2.5% compressibility ratios of reconstructed *x*–*z* slices from bright-field and dark-field tomograms are expressed as a multiple (denoted as *ρ*) of the 2.5% compressibility ratios of the membrane phantom. A distribution of *ρ* values is obtained for each of the 1024 membrane phantom *x*–*z* slices. The mean of ρ plus or minus one sample standard deviation is then plotted in each transform domain. Larger ρ values indicate a less sparse experimental *x*–*z* slice, and the minimum number of measurements required to reconstruct an image scales linearly with *ρ*. A dashed line at *ρ* = 1 is included in each figure as a point of reference. (**a**) *ρ* values in the TV domain, (**b**) *ρ* values in the identity domain, (**c**) ρ values in the DBS wavelet domain.

Our lack of prior knowledge about the experimental data structure complicates analysis of the dataset’s compressibility in any of the transform domains. Nevertheless, we can estimate compressibility by analyzing high-quality reconstructions obtained from a fully sampled tilt series across several relative compressibility thresholds. This is illustrated in Fig. 5, which displays *ρ*, the 2.5% compressibility ratios of the high-dose bright-field and dark-field datasets as a multiple of the 2.5% compressibility ratios of the membrane phantom. For each of the 1024 *x*–*z* slices of the experimental reconstruction, a distribution of *ρ* values is calculated relative to the compressibility obtained from the 256 membrane phantom slices; for each transform domain, the mean of these distributions plus or minus one standard deviation is plotted in Fig. 5a–c. The experimental datasets are significantly less compressible than the membrane phantom dataset in the TV and identity domains, whereas the opposite is true in the wavelet domain. This is likely due to the presence of sharp boundaries in the membrane phantom, leading to the slow decay of its wavelet coefficients. Regardless, while a wavelet regularization term does improve the experimental data reconstructions, even a large value is insufficient for regaining the CS-ET performance improvements exhibited in the phantom studies.

## Discussion

Previous work has established that, at least in some imaging applications, CS techniques allow for high-quality recovery of tomograms from highly undersampled data. The present study corroborates these results, and provides evidence that the quality of CS-ET matches or exceeds that of other recovery techniques for undersampled recovery of more complex images. Our results also challenge the notion that undersampling is necessarily an attractive goal for CS techniques applied to STEM tomography of biological specimens. From Figs 3 and 4 it is evident that even moderate undersampling creates structurally significant degradation of reconstruction resolution, likely a result of the decreased compressibility of the biological data. Therefore, we conclude that the optimal strategy for a microscopist constrained by the total electron dose on a beam-sensitive biological specimen is to fractionate the dose over a fully sampled data set, provided that the fractionated electron dose is sufficient to detect the fiducial gold nanoparticles used to align the individual projections. However, in the collection of cryo-tomographic tilt series, the electron dose can be severely restricted due to limitations of radiation damage, in which case the fiducial gold nanoparticles might not be detectable in each dose-fractionated projection. In cryo-tomography of thick specimen, the number of tilts needed to obtain a given spatial resolution using conventional WBP or SIRT reconstruction methods may therefore exceed the maximum allowed dose. Under such circumstances CS-ET might offer important advantages.

Although the performances of the three algorithms (CS-ET, SIRT, and WBP) are closely matched when applied to fully sampled biological tilt series acquired at high-dose, as illustrated in Fig. 3, the CS-ET algorithm appears to be more useful for improving reconstructions of noisy images under lower dose conditions, and appears to function as a regularized reconstruction method, as we demonstrated in Fig. 4.

Moreover, randomized sampling of projections in the feasible tilt angle range does not appear to provide a superior alternative to the conventional tilt series acquisition procedure, which uses a constant angular increment. This might be attributable to random clumping of the tilt angles when the total number of Radon transform projections is of the order of 70, which is typical in the acquisition of ET tilt series. Such clumping could result in wedges of missed sampling in the angular range of the tilt series. Moreover, this phenomenon can be related to the coherence of the Radon transform measurement vectors. Ideal CS applications involve incoherent (almost-uncorrelated) measurements, but Radon transform measurements at the same radial coordinate and proximal angular coordinates can be highly coherent. A uniform sampling procedure maximizes the minimum angular distance between successive projections, minimizing the worst-case coherence of the measurement system. However, this explanation remains intuitive and further work is required to establish whether it can be grounded properly in CS theory. Open questions remain in the mathematical underpinnings of the recovery of sparse images from both randomized and uniform tomographic tilt series, but our finding is contrary to the prevailing trends in existing CS application results, wherein random measurement ensembles are found to recovery sparse images with high probability. Therefore, in view of the present results, a more careful exploration of this randomized measurement variant by acquiring new randomized STEM tilt series does not seem warranted. The application of the CS-ET algorithm to fully sampled, uniform tilt series is thus not compressed sensing in the strictest definition of the term, and is better understood as a regularized least-squares recovery algorithm.

Regardless of the terminology used, the results of this paper show that optimization-based regularized image recovery algorithms taking advantage of prior structural knowledge can be usefully applied to experimental STEM tomographic datasets from biological specimens. CS-ET can thus offer performance competitive with or exceeding other common reconstruction algorithms. In this work, prior structural knowledge was incorporated in the form of the 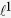 norm functions added to the optimization function in Equations (6 and 7). These encode prior assumptions about the sparsity of structure in the TV, identity, and wavelet domains. For example, cellular ultrastructure often predominantly comprises membranes that manifest as sparse one-dimensional line contours in the *x*-*z* plane, implying that the identity transform is an appropriate representation. Certain cell types can be packed with organelles like secretory granules that manifest as piecewise constant regions in the *x*-*z* plane, making the TV transform a suitable representation. Other structures such as chromatin within the cell nucleus neither have the intrinsically sparse representation of membranes nor the piecewise constant representation of secretory granules; these might best be represented by a wavelet transformation as included in Equation (7).

This paper focuses on the recovery of TV-, identity-, and wavelet-sparse tomograms, but there are many natural variations based on alternative sparsity models, which might be relevant to STEM imaging. Wavelet bases and frames have long been known to yield sparse representations of natural images, and recent developments in sparse imaging via anisotropic frame representations. For example, curvelets^38^,^39^, or shearlets^40^,^41^, may prove well suited to CS-ET. Data-driven representation methods are another possible avenue of development, tailored to dataset structure and learned during the reconstruction procedure. In this regard, Xu *et al*.^5^ have demonstrated a similar idea for CT, while Gopinath *et al*.^42^ have discussed a shape-based regularization procedure tailored to 3D tomogram structure.

Rather than decomposing the 3D reconstruction problem into 2D slices, the CS reconstruction theory is equally applicable to the recovery of 3D regions considered as a single domain. Currently, the memory requirements for large volumes prevent the creation of efficient fully 3D reconstruction algorithms, but that may change with the advance of computing technology. In terms of reconstruction quality, fully 3D sparse reconstructions are likely to be superior to their 2D equivalents, due to the added information on structural regularity supplied by the spatial relationships between the *x*–*z* slices.

Beyond these implementation details, the analysis in this paper confronts interesting and challenging open questions in the theory of compressed sensing. The recovery of sparse images from measurement matrices having the non-restricted isometry property (non-RIP matrices), such as those created by tomographic sampling, is still poorly-understood. Further research into understanding its empirically observed efficacy would shed valuable light on interpreting experimental measurement procedures from many imaging domains in the CS framework.

## Methods

### Experimental data

The three experimental datasets used in this paper are tomographic tilt series acquired from a stained beta cell in a plastic-embedded mouse pancreatic islet of Langerhans. The three tilt series were acquired on an FEI Tecnai TF30 TEM operating at 300 kV (FEI Inc., Netherlands). Images were acquired in the STEM mode, using a high-tilt tomography holder (Fischione Instruments, Inc., Export, PA), in conjunction with an on-axis bright field detector (Gatan Inc., Pleasanton CA) and in-column high-angle annular dark-field STEM detector (Fischione Instruments, Inc., Export, PA). For each dataset, a single tilt series was acquired automatically with a tilting range of ±78° and increments of approximately 2°, resulting in 79 projections. Each projection was 2,048 × 2,048 pixels, with a pixel size of 1.67 nm (BF) and 1.4 nm (DF). Two tilt series were acquired in the DF-STEM mode, one with a standard probe current of ~1 nA and one with a probe current of ~0.1 nA, to test the reconstruction algorithms under noisy conditions. The tilt series were aligned using IMOD software^43^ and binned by a factor of 2. An example of a binned projection is shown in Supplementary Fig. 4.

Each reconstructed volume of 1,024 independent reconstructions of 1,024 × 100 2D images (N = 102, 400). Figure 1a demonstrates this paper’s choice of orientation for the coordinate axes, consistent with common nomenclature in the ET community. Each reconstruction generates *x*–*z* data of the tomogram volume for a fixed y value, but the perspective most commonly used for examining imaged specimens is the orthogonal *x*–*y* view.

The use of the identity sparsity model with the experimental images requires background subtraction from the resulting reconstructions before evaluating the reconstruction sparsity. Background values were estimated by averaging over patches of manually observed background areas in an initial WBP reconstruction.

### Simulated data

Key differences between the imaging of biological relative to nanoscale metallurgical specimens are the much greater complexity of the 3D volumes, as well as decreased signal-to-noise ratio resulting from the lower electron dose. Tomograms of assemblies of nanoparticles may be highly sparse or TV-sparse compared with the decreased sparsity of biological tomograms. In addition to the evidence from experimental reconstructions for the decreased utility of undersampling for biological CS-ET, we consider the problem numerically by using a simulated nanometallurgical phantom shown in Supplementary Fig. S3. The construction of this phantom is intended to draw on the observed statistical properties of datasets^12^,^23^, e.g., high contrast and piecewise constant components with sharp edges. Projections of the nanoparticle phantom were simulated in MATLAB. Poisson noise with a rate parameter of 5,500 is added to the projections, which were then corrupted with Gaussian noise with a standard deviation equal to 10% of the projection’s mean.

The membrane phantom consists of a 256 × 100 × 256 array taking values in [0, 1]. On top of a non-zero background, multiple ellipses of random sizes, locations, eccentricities, orientations, and contrasts are superimposed to imitate observed arrangements of membranous bodies in heavy metal-stained cellular preparations. This phantom provides contrast and structural conditions more akin to those encountered in biological imaging, and sparsity levels between those found in the nanoparticle phantom and the experimental datasets. Noisy projections were generated by the addition of Poisson noise, to produce an average noise level of 10% of the projection’s mean after background subtraction, commensurate with that observed experimentally. Both noiseless and noisy projection data were tested.

### Reconstruction of experimental tilt series

The experimental datasets were reconstructed using CS-ET, SIRT, and WBP algorithms from 1×, 3×, and 6× projection data, with the bright-field datasets containing 79 tilts, 27 tilts, and 14 tilts, respectively, and the dark-field dataset containing 77, 26, and 13 tilts, respectively. Figures 3 and 4, as well as Supplementary Figs 4 and 5, illustrate the results of this procedure. After reconstructions were performed, all tomogram volumes were whitened (scaled to zero mean and given unit variance) and then uniformly scaled for better image contrast for visualization purposes.

All regularized reconstruction methods require a choice of hyperparameter weights, and many open problems remain for obtaining good *a priori* estimates of weight values in experimental application conditions. Parameter combinations used in this paper were found heuristically based on experience with the datasets. Leary *et al*.^9^ have discussed hyperparameter tuning concerns in more detail.

The sparsity of the experimental datasets was quantified using the n%-compressibility ratio for n = 2.5 represented as a distribution of multiples *ρ* of the 2.5%-compressibility ratios for the 256 *x*–*z* slices of the membrane phantom. This distribution gives an indication of the relative compressibility of the experimental and phantom datasets.

### CS-ET numerical implementation

All code used in this paper may be freely downloaded. The code is maintained at https://github.com/norbert-wiener-center/cset, Guay, M.D., A MATLAB library for compressed sensing (CS) reconstruction of electron tomogram (ET) volumes. Date of access: 05/05/16.

An archived copy is available at https://figshare.com/articles/cset_zip/2303917 (DOI 10.6084/m9.figshare.2303917), Guay, M., Czaja, W., Aronova, M., Leapman, R., CS-ET algorithm, MATLAB implementation. Date of access: 05/05/16.

The experimental datasets used in this paper are available at https://figshare.com/articles/CS_ET_Experimental_Datasets/2314630 (DOI 10.6084/m9.figshare.2314630), Guay, M., Czaja, W., Aronova, M., Leapman, R., CS-ET Experimental Datasets. Date of access: 05/05/16.

Equation (7) is a convex optimization problem, but the 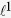-norm is not a globally differentiable function, which presents an obstacle to efficient optimization. In recent years, several efforts have been made to create algorithms for rapidly solving 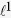-regularized least-squares problems. We choose to use the split Bregman method^44^, in contrast to the conjugate-gradient and interior-point methods used in prior studies^9^,^31^,^45^.

In addition to the *λ*_*TV*_*, λ*_*I*_, and *λ*_*W*_ regularization parameters in (7), the split Bregman method requires the specification of a data fidelity parameter *ν*. A convenient method of parameter specification is to fix a value of *ν* and write (*λ*_*TV*_ *, λ*_*I*_ *, λ*_*W*_) as multiples of the *ν* value. In this way, *ν* can be viewed as controlling the size of each update of the iterative split Bregman procedure. This method allowed us to make simple changes to the hyperparameter sets used for each level of undersampling in the experimental reconstructions.

The bright-field dataset used the following parameter values. For the 1× reconstruction, *ν*_*1*_ = 5 × 10^−6^. The 3× and 6× reconstructions had *ν*_3_ = *ν*_6_ = 1 × 10^−5^. All bright-field reconstructions had (*λ*_*TV*_ *, λ*_*I*_ *, λ*_*W*_) = *ν*_*i*_ · (1.2, 6, 4) for i = 1, 3, 6.

The dark-field dataset used different parameter sets. For all three reconstructions, *ν*_1_ = *ν*_3_ = *ν*_6_ = 10^−6^. Then, (*λ*_*TV,1*_ *, λ*_*I,1*_ *, λ*_*W,1*_) = *ν*_1_ · (4, 8, 10); (*λ*_*TV,3*_ *, λ*_*I,3*_ *, λ*_*W,3*_) = *ν*_3_ · (6, 2, 6); and (*λ*_*TV,6*_ *, λ*_*I,6*_ *, λ*_*W,6*_) = *ν*_6_ · (4, 2, 4).

As an iterative method the split Bregman method also requires the specification of a number of iterations, in this case divided into “inner” loop iterations and “outer” loop iterations. All experimental reconstructions were done with 12 inner loop iterations and 10 outer loop iterations.

Parameters in this study were chosen by visual inspection of 2D slices of full 3D reconstructions, selecting values that provided highest image contrast and minimized reconstruction artifacts. To the authors’ knowledge, there do not exist general, practical methods for *a priori* estimation of regularization hyperparameter values. However, once an appropriate value for *ν* is found, reconstructions are robust to small changes in the values of the {*λ*_*i*_} parameters, and values producing high-quality reconstructions were found quickly for all data sets.

We adapted the core CS-ET algorithm from the mriis.m MATLAB function developed by Goldstein and Osher^42^, by modifying the algorithm to use Radon measurement matrices instead of Fourier measurement matrices. This modification prevents us solving the intermediate 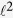-minimization problem using Fourier methods, and so we use a conjugate gradients implementation tailored to ASTRA’s Radon transform code.

Reconstruction of 3D volumes through independent 2D problems lends itself naturally to parallelization to improve reconstruction times. This was implemented in a simple way in MATLAB using the *parfor* loop structure, reducing the fully sampled reconstruction time to less than 20 minutes. The time required for the undersampled reconstructions was proportional to the level of undersampling: about 10 minutes for 2× undersampling and 7 minutes for 3× undersampling. Experimental dataset reconstruction was carried out on a Windows 7 PC with dual 8-core Intel^®^ 3.40 GHz CPU’s, using a Xeon®MATLAB parallel pool using 26 workers. All GPU calculations were performed on a single NVIDIA Tesla^®^ K20c GPU.

### Comparisons between reconstruction methods

Experimental dataset CS-ET reconstructions were compared against WBP and SIRT reconstructions of the same projections. WBP reconstructions were implemented in MATLAB using the MATLAB Imaging Toolbox’s *iradon* command. SIRT reconstructions were written in MATLAB and Fortran and run on the high-performance Biowulf Linux cluster at the National Institutes of Health (http://biowulf.nih.gov). Simulated dataset CS-ET reconstructions were compared against WBP reconstructions performed in MATLAB with the imaging toolbox’s *iradon* command.

### Reconstruction of simulated tilt series

The goal of reconstructing the nanoparticle phantom was to establish a baseline comparison between our CS-ET implementation and the Fourier-based method described by Leary *et al*.^9^. The full set of data analyzed in this work supports a conclusion that CS-ET does not substantially outperform alternative methods such as SIRT and WBP for less-compressible signals, so we seek to eliminate algorithm implementation errors as a source of performance degradation. A description of the nanoparticle phantom reconstruction procedure is described in Supplementary Fig. S3.

For the membrane phantom, reconstructions were analyzed in a standard manner via their root mean squared error: 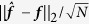, where *N* = 6, 553, 600 is the number of voxels in the phantom dataset, calculated for each 2D *x*–*z* phantom slice ***f***. The root mean square error (RMSE) was calculated for all reconstructions, and the CS-ET results were compared for reconstructions performed with the identity, wavelet, and TV sparsity transforms for each slice of the 3D phantom to examine the correlation between them. This analysis is presented in Supplementary Fig. S5.

Reconstructions were computed for the membrane phantom using both random and conventional tomographic sampling schemes (random and uniformly spaced tilt angles, respectively), to test the performance of the randomized sampling strategy. The uniform tilt angles were chosen to coincide with the tilt series angles that we used to acquire experimental bright-field tomographic datasets. Random tilt angle vectors were chosen to have the same number of projections as the uniform series, chosen at random from the same tilt range as the uniform angles. The results of this analysis are displayed in Fig. 2d,e.

To test the stability of the reconstruction algorithm, all reconstructions were repeated under two different noise conditions. First, reconstructions were generated from projection data free of noise. Second, reconstructions were generated from projections corrupted by Poisson noise with a rate parameter of 5500 and with standard deviation equal to 10% of the projection mean. Since the noisy conditions are more relevant to experimental measurement conditions, images of those results were included in Fig. 1, whereas the noiseless reconstruction is presented in Supplementary Fig. S4.

## Additional Information

**How to cite this article**: Guay, M. D. *et al*. Compressed Sensing Electron Tomography for Determining Biological Structure. *Sci. Rep.* **6**, 27614; doi: 10.1038/srep27614 (2016).

## Supplementary Material

Supplementary Information

## References

[b1] Lustig M., Donoho D. & Pauly J. Sparse MRI: the application of compressed sensing for rapid MR imaging. Magn. Reson. Med. 58(6), 1182–1195 (2007). 17969013 10.1002/mrm.21391

[b2] Cloninger A., Czaja W., Bai R. & Basser P. Solving 2D Fredholm integral from incomplete measurements using compressive sensing. SIAM Journal on Imaging Sciences 7(3), 1775–1798 (2014). 10.1137/130932168 PMC8279431 34267858

[b3] Bai R., Cloninger A., Czaja W. & Basser P. Efficient 2D MRI relaxometry using compressed sensing. J. Magn. Reson. 255, 88–99 (2015). 25917134 10.1016/j.jmr.2015.04.002

[b4] Haftka A., Celik H., Cloninger A., Czaja W. & Spencer R. 2D sparse sampling algorithm for ND Fredholm equations with applications to NMR relaxometry. *IEEE Conference Publications*, SampTA 2015: Sampling Theory and Applications, American University, Washington, DC, 367-371 (2015).

[b5] Xu Q. . Low-dose x-ray CT reconstruction via dictionary learning. IEEE Transactions on Medical Imaging 31(12), 1682–1697 (2012). 22542666 10.1109/TMI.2012.2195669 PMC3777547

[b6] Saghi Z. . Three-dimensional morphology of iron oxide nanoparticles with reactive concave surfaces. a compressed sensing-electron tomography (CS-ET) approach. Nano letters 11(11), 4666–4673 (2011). 21950497 10.1021/nl202253a

[b7] Binev P. . Compressed sensing and electron microscopy. In: Modeling Nanoscale Imaging in Electron Microscopy Springer, pp. 73–126 (2012).

[b8] Goris B., Van den Broek W., Batenburg K. J., Mezerji H. H. & Bals S. Electron tomography based on a total variation minimization reconstruction technique. Ultramicroscopy 113, 120–130 (2012).

[b9] Leary R., Saghi Z., Midgley P. A. & Holland D. J. Compressed sensing electron tomography. Ultramicroscopy 131, 70–91 (2013). 23834932 10.1016/j.ultramic.2013.03.019

[b10] Saghi Z. . Compressed sensing electron tomography of needle-shaped biological specimens – Potential for improved fidelity with reduced dose. Ultramicroscopy 160, 230–238 (2016). 26555323 10.1016/j.ultramic.2015.10.021

[b11] Aganj L. . Regularization for inverting the Radon transform with wedge consideration. In: 4th IEEE International Symposium on Biomedical Imaging: Macro to Nano Vols 1–3, IEEE, New York, pp. 217–220 (2007).

[b12] Song K., Comolli L. & Horowitz M. Removing high contrast artifacts via digital inpainting in cryo-electron tomography: An application of compressed sensing. J. Struct. Biol. 178(2), 108–120 (2012). 22248454 10.1016/j.jsb.2012.01.003

[b13] Vogel C. & Oman M. Iterative methods for total variation denoising. SIAM Journal on Scientifc Computing 17(1), 227–238 (1996).

[b14] Neumaier A. Solving ill-conditioned and singular linear systems: a tutorial on regularization. SIAM Review 40(3), 636–666 (1998).

[b15] Walnut D. An introduction to wavelet analysis. Springer, New York (2002).

[b16] Hugel M., Rauhut H. & Strohmer T. Remote sensing via *l*_1_ minimization. Foundations of Computational Mathematics 14(1), 115–150 (2014).

[b17] Frank J. Electron tomography: Methods for Three-dimensional Visualization of Structures in the Cell. Springer, New York (2006).

[b18] Koster A. J. . Perspectives of molecular and cellular electron tomography. J. Struct. Biol. 120, 276–308 (1997). 9441933 10.1006/jsbi.1997.3933

[b19] Milne J. L. & Subramaniam S. Cryo-electron microscopy of bacteria: progress, challenges and future prospects. Nature Rev. Microbiol. 7(9), 666–675 (2009). 19668224 10.1038/nrmicro2183 PMC6993139

[b20] Grünewald K., Desai P., Winkler D. C., Belnap D. M. & Steven A. C. Three-dimensional structure of herpes simplex virus from cryo-electron tomography. Science 301(5649), 1396–1398 (2003). 14631040 10.1126/science.1090284

[b21] McIntosh J. R., Nicastro D. & Mastronarde D. New views of cells in 3D: an introduction to electron tomography. Trends Cell Biol. 15(1), 43–51 (2005). 15653077 10.1016/j.tcb.2004.11.009

[b22] Marsh B. J., Mastronarde D. N., Buttle K. F., Howell K. E. & McIntosh J. R. Organellar relationships in the Golgi region of the pancreatic beta cell line, HIT-T15, visualized by high resolution electron tomography. Proc. Natl. Acad. Sci. USA 98(5), 2399–2406 (2001). 11226251 10.1073/pnas.051631998 PMC30150

[b23] Hohmann-Marriott M. F. . Nanoscle 3D cellular imaging by axial scanning transmission electron tomography. Nature Methods 6(10), 729–731 (2009). 19718033 10.1038/nmeth.1367 PMC2755602

[b24] Yakushevska A. E. . STEM tomography in cell biology. J. Struct. Biol. 159, 381–391 (2007). 17600727 10.1016/j.jsb.2007.04.006

[b25] Sousa A. A., Azari A., Zhang G. F. & Leapman R. D. Dual-axis electron tomography of biological specimens: extending the limits of specimen thickness with bright-field STEM imaging. J. Struct. Biol. 174(1), 107–114 (2011). 21055473 10.1016/j.jsb.2010.10.017 PMC3056916

[b26] Radermacher M. Weighted back-projection methods. In: Electron tomography pp. 245–273 Springer, New York (2006).

[b27] Kak A. C. & Slaney M. Principles of computerized tomographic imaging, IEEE Press, New York (1988).

[b28] Donoho D. & Huo X. Uncertainty principles and ideal atomic decomposition. IEEE Transactions on Information Theory 47(7), 2845–2862 (2001).

[b29] Candes E. J. & Romberg J. Sparsity and incoherence in compressive sampling. Inverse Problems 23(3), 969–985 (2007).

[b30] Sima D. *Regularization techniques in model fitting and parameter estimation*. Ph.D. Thesis, Katholieke Universiteit Leuven, Leuven, Belgium (2006).

[b31] Goris B., Roelandts T., Batenburg K. J., Mezerji H. H. & Bals S. Advanced reconstruction algorithms for electron tomography: from comparison to combination. Ultramicroscopy 127, 40–47 (2013). 22951262 10.1016/j.ultramic.2012.07.003

[b32] Daubechies I. Orthonormal bases of compactly supported wavelets. Communications on Pure and Applied Mathematics 41(7), 909–996 (1988).

[b33] Gottlieb D., Gustafsson B. & Forssen P. On the direct Fourier method for computer tomography. IEEE Transactions on Medical Imaging 19(3), 223–232 (2000). 10875706 10.1109/42.845180

[b34] Fessler J. A. & Sutton B. Nonuniform fast Fourier transforms using min-max interpolation. IEEE Transactions on Signal Processing 51(2), 560–574 (2003).

[b35] Matej S., Fessler J. & Kazantsev I. Iterative tomographic image reconstruction using Fourier-based forward and back-projectors. IEEE Transactions on Medical Imaging 23(4), 401–412 (2004). 15084066 10.1109/TMI.2004.824233

[b36] Bracewell R. N. The Fourier transform and its applications, Vol. 31999 McGraw-Hill, New York (1986).

[b37] Cai T. . Deletion of IA-2 and/or AI-2β in mice decreases insulin secretion by reducing the number of dense core vesicles. Diabetologia 54(9), 2347–2357 (2011). 21732083 10.1007/s00125-011-2221-6 PMC3168514

[b38] Candes E., Demanet L., Donoho D. & Ying L. Fast discrete curvelet transforms. Multiscale Modeling and Simulation 5(3), 861–899 (2006).

[b39] De Hoop M. V., Smith H., Uhlmann G. & Van der Hilst R. D. Seismic imaging with the generalized Radon transform: a curvelet transform perspective. Inverse Problems 25(2), 025005 (21 pp.) (2009).

[b40] Kutyniok G. & Labate D. In: Shearlets: Multiscale Analysis for Multivariate Data. Springer Science and Business Media (2012).

[b41] Colonna F., Easley G., Guo K. H. & Labate D. Radon transform inversion using the shearlet representation. Applied and Computational Harmonic Analysis 29(2), 232–250 (2010).

[b42] Gopinath A. . Shaped-based regularization of electron tomographic reconstruction. IEEE Transactions on Medical Imaging 31(12), 2241–2252 (2012). 22922711 10.1109/TMI.2012.2214229 PMC3513577

[b43] Kremer J. R., Mastronarde D. N. & McIntosh J. R. Computer visualization of three-dimensional image data using IMOD. J. Struct. Biol. 116(1), 71–76 (1996). 8742726 10.1006/jsbi.1996.0013

[b44] Goldstein T. & Osher S. The split Bregman method for L1-regularized problems. SIAM Journal on Imaging Sciences 2(2), 323–343 (2009).

[b45] Candes E. J. & Romberg J. L1-magic: recovery of sparse signals via convex programming. Technical Report, Caltech, URL http://users.ece.gatech.edu/rjustin/l1magic/downloads/l1magic.pdf (2005).

