# Supplementary material for: Compressed Sensing Electron Tomography for Determining Biological Structure
Source: Sci Rep. 2016 Jun 13;6:27614. doi: 10.1038/srep27614 (PMC4904377; doi:10.1038/srep27614)
Supplement: Supplementary Information [file srep27614-s1.pdf]

Compressed Sensing Electron Tomography for Determining Biological Structure

Matthew D. Guay[1§], Wojciech Czaja[2], Maria A. Aronova[3], Richard D. Leapman[3†]

[1]University of Maryland, Department of Applied Mathematics and Scientific Computation, College Park, MD 20742, USA

[2]University of Maryland, Department of Mathematics, College Park, MD 20742, USA

[3]National Institute of Biomedical Imaging and Bioengineering, National Institutes of Health, Bethesda, MD 20892, USA

Keywords: Electron tomography, 3D reconstruction, compressed sensing, regularization methods, cellular ultrastructure.

Correspondence to:

[§]Matthew D. Guay, University of Maryland, Department of Applied Mathematics and Scientifc Computation, 1301 Mathematics Bldg., College Park, Maryland 20742, USA. E-mail: mguay@math.umd.edu

[†]R.D.Leapman, NIBIB, National Institutes of Health, Bldg. 13, Rm. 3N17, 13 South Drive, Bethesda, MD 20892, USA. Tel: 301-496-2599; e-mail: leapmanr@mail.nih.gov

Fig. S1

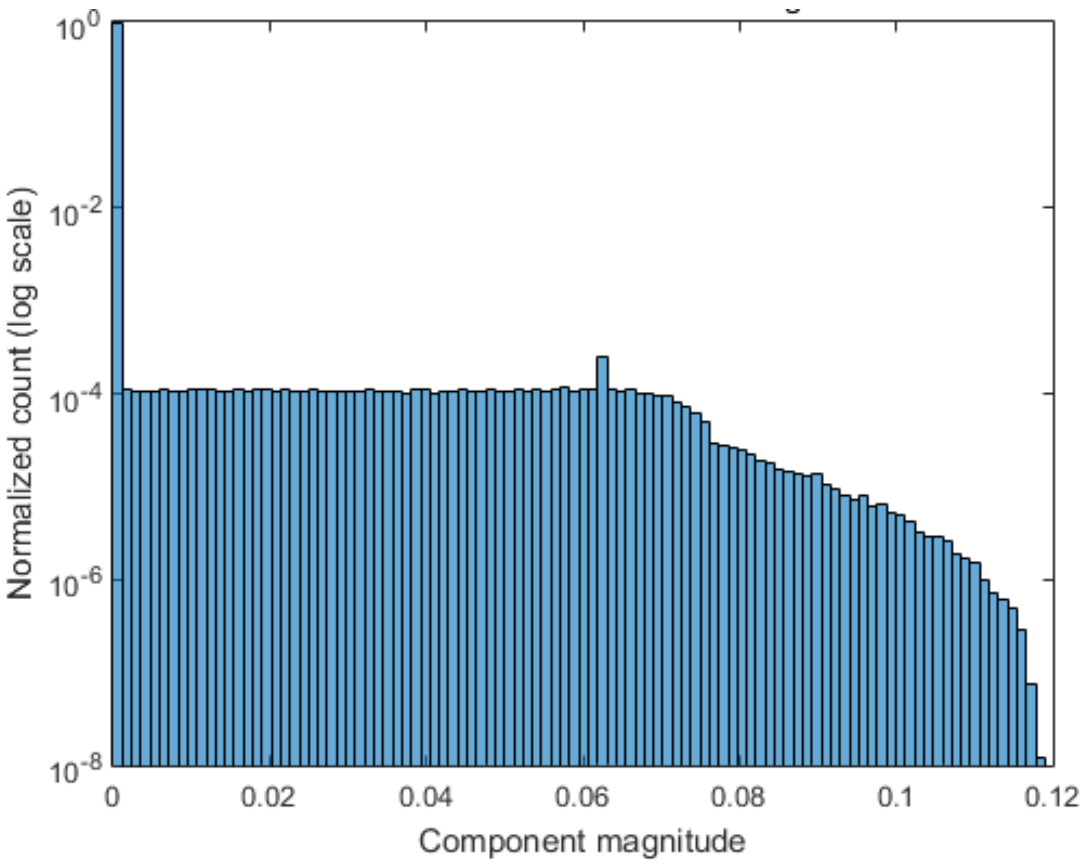

Supplementary Figure S1

Estimate of coherence of a Radon Transform measurement dictionary. A histogram of the magnitudes of the components of Radon transform measurement vectors, taken from a Radon transform of a 256×256 image at angles from $-70°$ to $+70°$ at $5°$ increments. The coherence of a measurement dictionary containing vectors of length M is defined as $\sqrt{M}$ times the largest magnitude among the vectors' components, and is used as a convenient but conservative bound for the restricted isometry property (RIP) of a measurement system. For the measurement dictionary displayed here, consisting of 7424 length-65536 vectors, the coherence value is approximately $0.1188 \cdot 256 = 30.4$. This value is too large to be of use for a theoretical analysis, suggesting the need for new analytic tools for real-world measurement systems.

Fig. S2

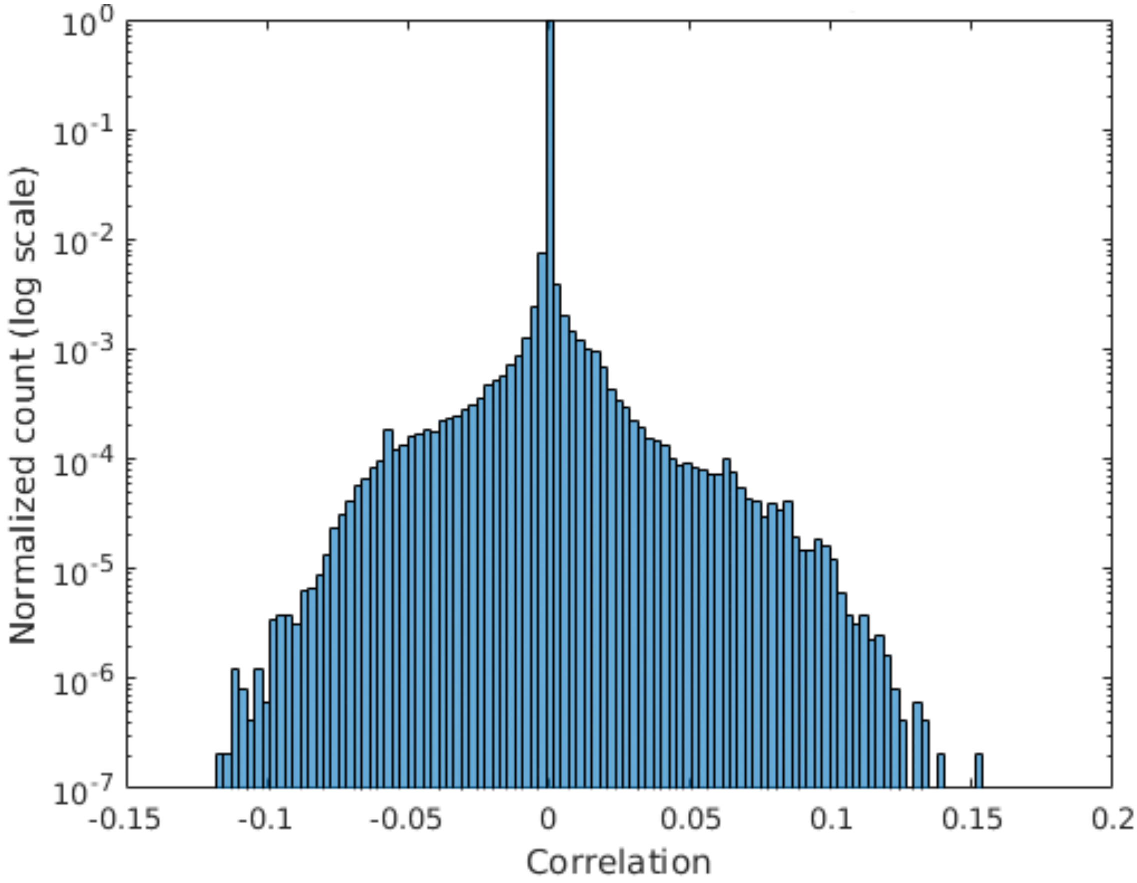

Supplementary Figure S2

Estimate of mutual coherence between Radon Transform measurement dictionary and DB8 wavelet representation. A histogram of the correlation values between normalized discrete Radon transform measurement vectors and elements of a DB8 wavelet basis, each for a 256 × 256 image and sampling 10% of the vectors in each dictionary. The Radon transform used consists of projections at angles from −70° to +70° at 5° increments. The mutual coherence of a measurement dictionary and representation basis for vectors of length M is defined as the M times the magnitude of the largest correlation between their elements, and is used as an upper bound for the restricted isometry property (RIP) of a measurement system for signals with sparse representations in the given representation basis. For the dictionaries displayed here, a mutual coherence of approximately $0.15 \cdot 256 = 38.4$ is too large to be of use to a theoretical analysis of the optimization routine, suggesting the need for new analytic tools for real-world measurement systems.

Fig. S3

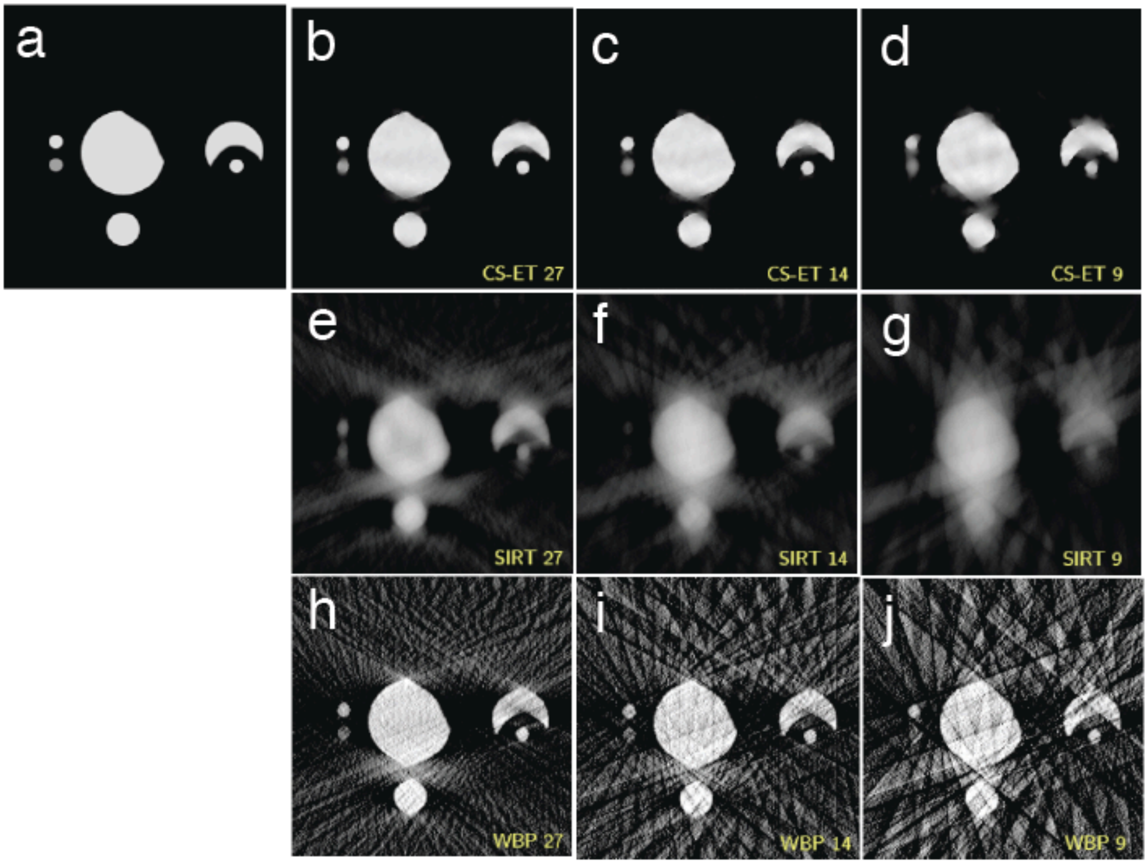

Supplementary Figure S3

Comparison of reconstruction techniques for undersampled recovery of an inorganic nanoparticle phantom. Simulated reconstructions from fully sampled tilt series containing 27 projections distributed over an angular range of ±65° with a 5° tilt increment, and 2× undersampled and 3× undersampled data containing 15, and 9 projections respectively. **(a)** Simulated mass distribution in $x-z$ plane from a single-phase nanoparticle phantom showing particles with piecewise constant density; **(b-d)** CS-ET reconstructions; **(e-d)** SIRT reconstructions; and **(h-j)** WBP reconstructions. Note the higher performance of CS-ET reconstruction for this simple, sparse model, in agreement with the earlier work of Leary et al. (2013).

Fig. S4

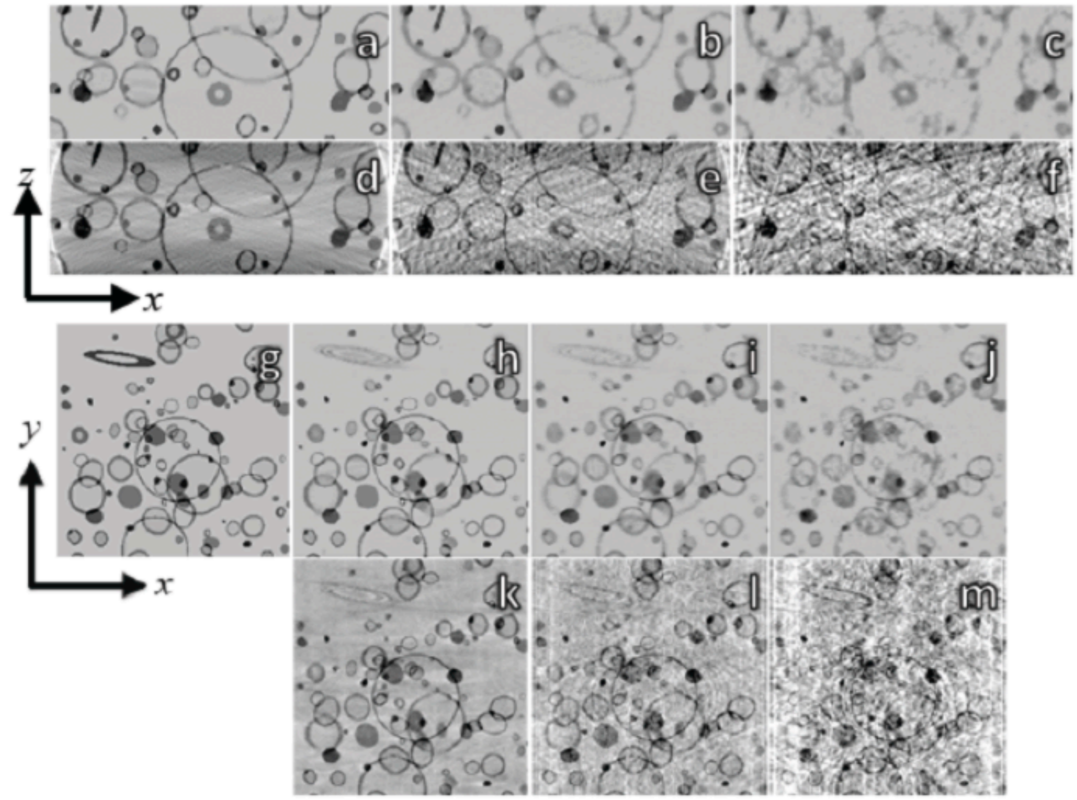

Supplementary Figure S4

CS-ET and WBP reconstructions from noiseless membrane phantom projections. The figure layout is identical to Fig. 1, but all reconstructions are shown from noiseless projection data. As expected, CS-ET substantially outperforms WBP and reconstruction quality is higher than that from noisy data. CS-reconstructed $x-z$ slices from **(a)** simulated projections at ±70° with 2° angular increment, **(b)** with 3× undersampling of tilt angles, and **(c)** 6× undersampling of tilt angles; WBP-reconstructed $x-z$ slices from **(d)** fully sampled tilt series, **(e)** with 3× undersampling of tilt angles, and **(f)** 6× undersampling of tilt angles; CS-reconstructed $x-y$ slices from **(g)** fully sampled tilt series, **(h)** with 3× undersampling of tilt angles, and **(i)** with 6× undersampling of tilt angles; WBP-reconstructed $x-y$ slices from **(j)** fully sampled tilt series, **(k)** with 3× undersampling of tilt angles, and **(l)** with 6× undersampling of tilt angles.

Fig. S5

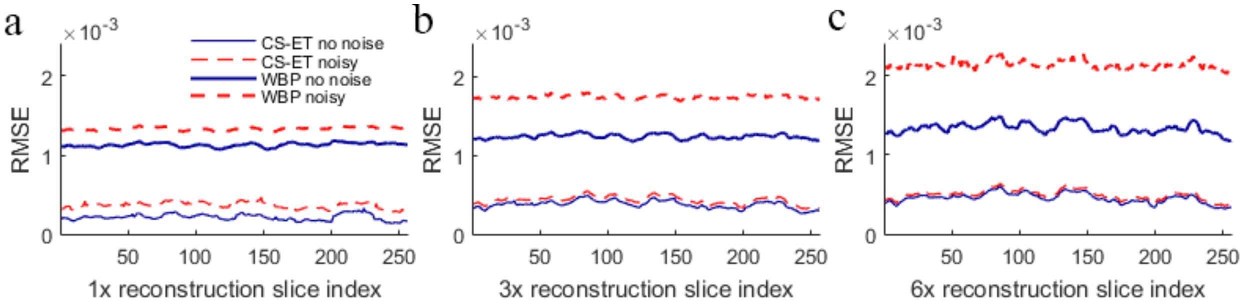

Supplementary Figure S5

Root mean square error (RMSE) for x−z slices from membrane phantom reconstruction. Values are displayed for CS-ET reconstructions (thin lines) and WBP reconstructions (thick lines) from noiseless (solid blue lines) and noisy (red dashed lines). **(a)** Fully sampled tilt series; **(b)** 3× undersampled tilt series; and **(c)** 6× undersampled tilt series. It is evident that the CS-ET reconstructions consistently outperform their WBP counterparts in RMSE value. Moreover, CS-ET reconstruction volumes are more robust to the addition of noise, with smaller relative and absolute increases in error values than those observed with WBP.

Fig. S6

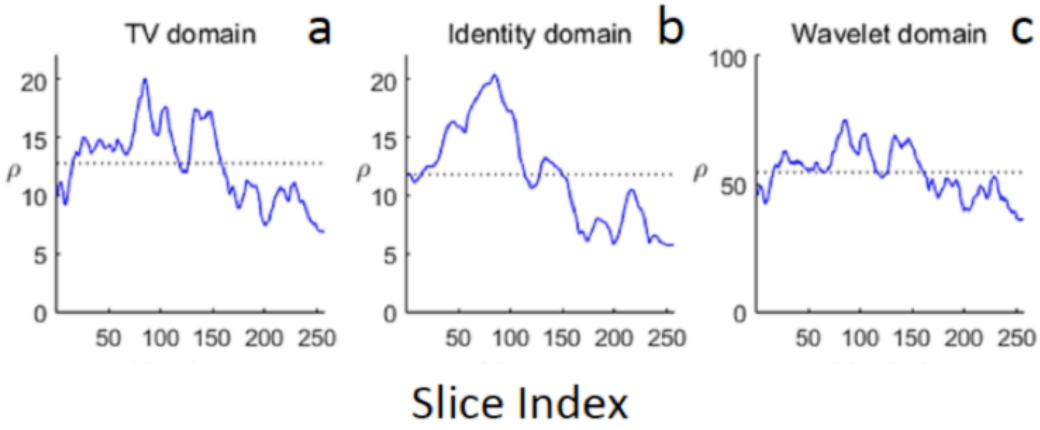

Supplementary Figure S6

Sparsity comparison for the membrane and nanoparticle phantoms. The 1%-compressibility ratios of each of the 256 $x-z$ slices of the membrane phantom are calculated in each transform domain and plotted as a multiple ρ of the nanoparticle phantom's 1%-compressibility ratio. **(a)** ρ in the TV domain has average value of 12.7; **(b)** ρ in the identity domain has average value of 11.5; **(c)** ρ in the DB8 wavelet domain has average value of 54.4. Although the membrane phantom has significantly simpler structure than is present in experimental biological datasets, it is markedly less compressible than the nanoparticle phantom.

Fig. S7

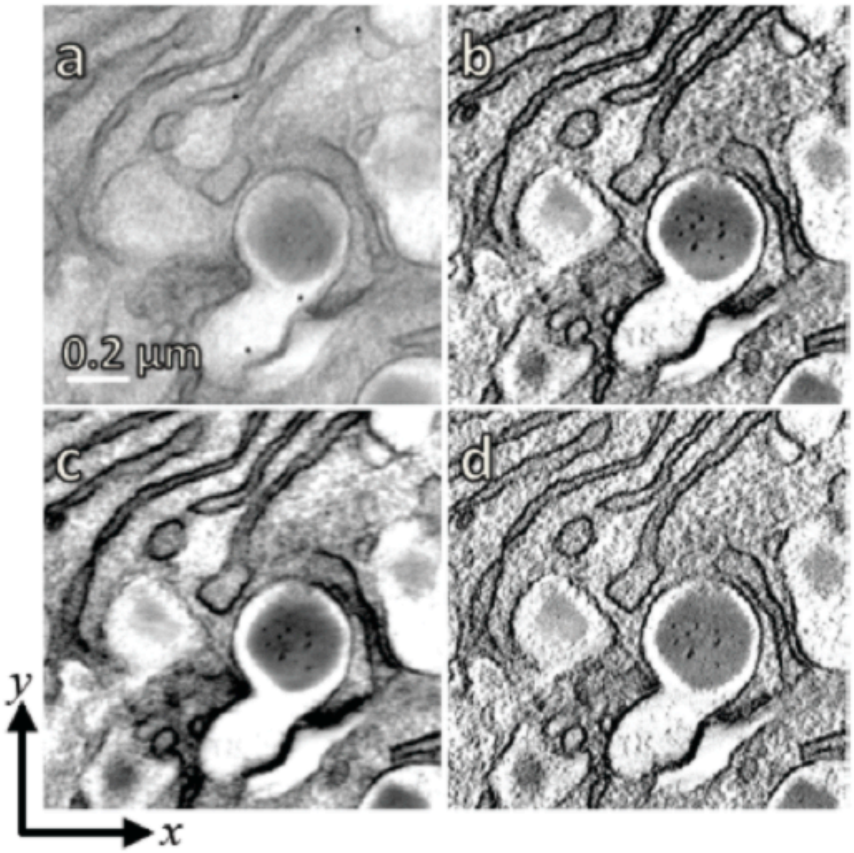

Supplementary Figure S7

Comparison of bright-field STEM projections and *x*−*y* reconstruction slices. (a) The STEM projection of a biological sample at 0° tilt after initial preprocessing in IMOD, which provides a reference for the full reconstructed volume. Typical *x*−*y* slices from fully sampled data are displayed in **(b)** for CS-ET reconstruction, **(c)** for SIRT reconstruction, and **(d)** for WBP reconstruction.

Fig. S8

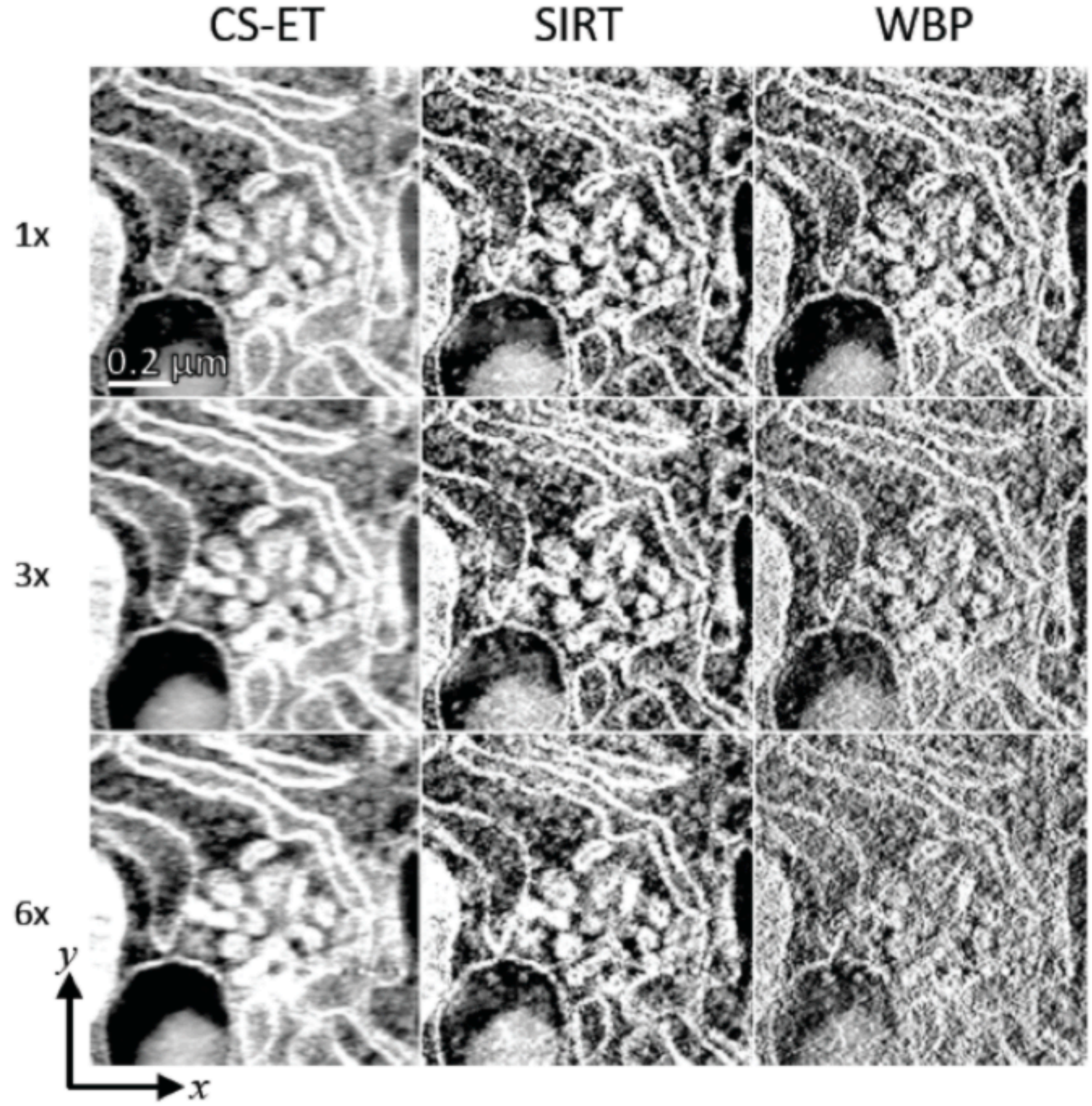

Supplementary Figure S8

Comparison of reconstructions from dark-field STEM tomographic tilt series, showing *x-y* othoslice through stained pancreatic beta cell. Vertical columns of images correspond to CS-ET, SIRT, and WBP reconstructions, and horizontal rows of images correspond to fully sampled, 3× undersampling, and 6× undersampling, as indicated.
